# Prevalence and factors associated with uncontrolled hypertension among adult hypertensive patients on follow-up at Northern Ethiopia, 2019: cross-sectional study

**DOI:** 10.11604/pamj.2020.36.187.23312

**Published:** 2020-07-15

**Authors:** Woldu Aberhe, Teklewoini Mariye, Degena Bahrey, Kidane Zereabruk, Abrha Hailay, Guesh Mebrahtom, Kibrom Gemechu, Brhanu Medhin

**Affiliations:** 1 School of Nursing, College of Health Sciences and Comprehensive Specialized Hospital, Aksum University, Tigray, Ethiopia,; 2 College of Health Sciences and School of Nursing, Adigrat University, Tigray, Ethiopia,; 3 College of Health Sciences and School of Nursing, Samara University, Samara, Afar, Ethiopia

**Keywords:** Ethiopia, prevalence, uncontrolled hypertension

## Abstract

**Introduction:**

uncontrolled hypertension is a major risk factor for cardiovascular, renal, and cerebrovascular morbidities and mortalities. This study aims to assess the prevalence and factors associated with uncontrolled hypertension among adult hypertensive patients.

**Methods:**

hospital-based cross-sectional study was conducted. Systematic random sampling technique was used to select 396 hypertensive patients. Respondents were interviewed and their medical charts were reviewed using pretested structured questionnaire. Bivariable logistic regression was employed to examine the crude associations between the outcome variable and determinant variables. This was followed by multivariable logistic regression analysis using those variables with P-value ≤ 0.25 in the bivariable analysis.

**Results:**

of the total 396 hypertensive patients the prevalence of uncontrolled hypertension was found to be 48.6%. One fourth (26.1%), 231(59.1%), 289(73.9%), and 151(38.6%) hypertensive respondents were non adherent to anti-hypertensive medication, physical exercise, low salt diet, and weight management respectively. Age ≥50 years old (AOR = 2.33, 95%CI: 1.25, 4.35), non-adherence to anti-hypertensive medication, (AOR = 1.82 95%CI: 1.08, 3.04), non-adherence to physical exercise (AOR = 1.79 95%CI: 1.13, 2.83), non-adherence to low-salt diet (AOR = 1.98 95%CI: 1.18,3.31), and non-adherence to weight management (AOR = 2.06, 95%CI: 1.31, 3.23) were significantly associated with uncontrolled hypertension.

**Conclusion:**

the prevalence of uncontrolled hypertension was high. Older hypertensive patients, non-adherent to their medications, physical inactivity, non-adherent to low salt diet and non-adherent to weight management were more likely to have uncontrolled hypertension. Therefore, more effort should be dedicated to those identified modifiable risk factors to maximize blood pressure control.

## Introduction

Uncontrolled hypertension is a major public health challenge among hypertensive patients both in high and low-income countries [1-4]. Globally, nearly 1 billion individuals are living with uncontrolled hypertension [5] with a proportion of 66.8% and 61.6%, in developed and developing countries respectively [6]. In SSA, hypertension affects about 25% of the adult population and the prevalence of uncontrolled hypertension is around 70% [7]. Despite Ethiopia was signed to achieve sustainable development goal to reduce the premature death from non-communicable diseases by one third from 2016 to 2030 but the annual death of Ethiopia population due to non-communicable disease such as uncontrolled hypertension was still high (39%) [8, 9]. Few studies in Ethiopia reported that the prevalence of uncontrolled hypertension ranges from 37-63% [10-13]. Uncontrolled hypertension is a major risk factor for cardiovascular, renal, and cerebrovascular morbidities and mortalities [14-16]. Cardiovascular-related morbidity and mortality is the most common adverse outcome of uncontrolled hypertension, which is responsible for occurrence of stroke, ischemic heart disease, peripheral arterial disease, aortic aneurysm and congestive heart failure [17, 18]. Like many other chronic non-communicable diseases, the prevalence of uncontrolled hypertension in Ethiopia is rising due to increased risk factors [19]. Evidence had shown that age, sex, non-adherence to antihypertensive medications, non-adherence to low salt intake, physical inactivity, and the number of medications and presence of comorbid disease are among the major contributing factors to uncontrolled hypertension [20-23]. Identifying these determinant factors are important to reduce uncontrolled hypertension among hypertensive patients. Therefore, this study aims to assess the prevalence of uncontrolled hypertension and its associated factors among hypertensive patients on treatment follow-up at Mekelle public hospitals.

## Methods

**Study area and period:** the study was conducted at Mekelle public hospitals of the Tigray region, Northern Ethiopia. Mekelle is found at 783 Km away to the north from the capital city of Addis Ababa. In Mekelle city, there are two general public hospitals (Mekelle and Quiha hospital), one comprehensive specialized hospital Ayder comprehensive specialized hospital that provides service for more than 9 million populations in its catchment areas. The study was conducted from March to May 2019 at Mekelle public hospitals.

**Study design:** a hospital-based cross-sectional study was conducted.

**Study population:** the study population was all adult hypertensive patients who were under treatment follow-up at Mekelle public hospitals during the data collection period.

**Inclusion and exclusion criteria:** all adult (≥ 18 years old) hypertensive patients who were on anti-hypertensive treatment follow up for at least 6 months duration at the time of data collection were included in this study. However, unconscious hypertensive patients and pregnant mothers were excluded from this study.

**Sample size determination:** the sample size was calculated using a single population proportion formula by considering the following assumptions:

$$n=Z_{\alpha /2}{}^{2}*p(1-p)/d^{2}$$

n = minimum sample size, P= estimated proportion of uncontrolled hypertension (57.1%) [24], d = the margin of sampling error (5%), and Z a/2 = is the standard normal variable at 1-a% confidence level (95%=1.96) n = ((1.96)^2^x 0.571(1-0.571)) / (0.05)^2^ = 377. By adding 5% of none response rate the final sample size was 396.

**Sampling technique and procedure:** from the two month report of 842 hypertensive patients who had attended follow up at Mekelle city public hospitals, the sample size was proportionally allocated to the three public hospitals. The first study subjects were randomly selected using the lottery method from each hypertension follow up unit of the three public hospitals. Finally, 396 hypertensive patients were selected using a systematic random sampling technique (K=2).

**Data collection tools and instrument:** a structured questionnaire was used to collect data from the selected study participants. Weight and height were measured to determine anthropometric data using the Seca weighing scale and stadiometer respectively. The tool contains four sections (socio-demographic, knowledge, behavioral and clinical) characteristics. The first part (socio-demographic) and the third (clinical characteristics) part of the tool were developed based on a review of different literatures Whereas Adherence to self-care activities (medication adherence, low salt diet, alcohol, and smoking) were measured using the H-SCALE [25].

**Data collection procedure:** five BSc nurses as data collectors and one senior BSc nurse as supervisor were recruited. The data were collected through face-to-face interviews and document reviews. Weight and height were measured with participants standing without shoes and wearing light clothing. Participants were standing upright with the head in the Frankfort plane for height measurement. Bodyweight (kg) was measured using an electronic scale to the nearest 10g, and standing height was measured using a wall stadiometer to the nearest 0.1cm. BMI was calculated as body weight (kg)/height (m^2^). The subjects were then classified into four WHO BMI cut-offs points. Underweight < 18.50, normal range 18.5-24.9, overweight 25-30, and obese ≥ 30s.

**Study variables:** the dependent variable is uncontrolled hypertension. The independent variables were socio-demographic characteristic (age, sex, marital status, religion, ethnicity, occupation, educational status, and residence), knowledge, behavioral characteristics (anti-hypertension medication adherence, low salt diet adherence, physical activity status, alcohol status, smoking status) and clinical characteristics (duration of hypertension, family history of hypertension, availability of BP cuff at home, BP monitoring at home or any else, co-morbidity, BMI status, number of anti-HTN drugs).

### Operational definition

**Adherent to low salt diet:** twelve items were assessed practices related to eating a healthy diet, avoiding salt while cooking and eating, and avoiding foods high in salt content. Scores of 6 or better were considered adherent [25].

**Diabetic mellitus:** was defined as self-reported diabetes or the use of hypoglycemic agents or both.

**Hypertension:** was defined as those who had a documented diagnosis of hypertension (i.e. BP ≥ 140/90 mmHg) or those on anti-hypertensive agents.

**Uncontrolled hypertension:** is BP ≥ 140/90 mmHg using digital sphygmomanometer for adult hypertensive clients without diabetes mellitus and chronic kidney disease for at least three consecutive follow-up measurements and blood pressure ≥ 130/80 mmHg using digital sphygmomanometer for adult hypertensive clients with diabetes mellitus and chronic kidney disease for at least three consecutive follow-up measurements.

**Physical activity:** was assessed by 2 items. How many of the past 7 days did you do at least 30 minutes total physical activity? and how many of the past 7 days did you do a specific exercise activity (such as swimming, walking or biking) other than what you do around the house or as part of your work? Responses were summed (range, 0-14). Participants who scored ≥ 8 were coded as adhering to physical activity recommendations [25].

**Weight management:** ten items were used to assess activities undertaken in the past 30 days to manage weight through dietary practices and physical exercise. Response categories ranged from strongly disagree to strongly agree. Participants who agree or strongly agree with all 10 items (score ≥ 40) were considered to be following good weight management practices [25].

**Data quality assurance:** to assure the data quality training was given for the data collectors and supervisor. The weight measured by the digital scale was checked that it was at zero before each measurement. The questionnaire was translated into the local language (Tigrigna) and back to English. Five percent of the questionnaire was pre-tested before data collection to check logical sequence and consistency with desired objectives at Adigrat general hospital. After completing the pre-test they were asked about the clarity and relevance of each item and then based on comments and responses collected during the pre-test, adjustments were made to the questionnaire. The supervisor and principal investigator supervised the correct implementation of the data collection procedure, checked the completeness and logical consistency of the study tool daily. Besides this, the principal investigator carefully checked the entered data and thoroughly cleaned it before the start of the analysis.

**Data processing and analysis:** the data were checked for completeness, the response was coded and entered into Epi-data manager version 4.4.3.1 for windows and exported to SPSS version 23 for analysis. Descriptive statistics were computed and the result was summarized and presented by texts, tables, percentage, and frequency. Mean and the standard deviation were used for normally distributed data. Analysis using bivariable logistic regression model was made to see the association between the explanatory variables and the outcome variable. This was followed by multivariable logistic regression analysis using those variables with P-value ≤ 0.25 in the bivariable analysis and statistical significance was declared at P < 0.05. The Magnitude of the association was measured by using the adjusted odds ratio at a 95% confidence interval. Hosmer - Lemeshow test (0.741) was used to check the fitness model. Multi-Collinearity was checked using the variance inflation factor (< 1.16) and tolerance test (> 0.86).

## Results

**Socio-demographic characteristics:** in the study, 391 hypertensive respondents had participated with a response rate of 98.7%. The mean age of the study participants was 52.5 years (± 12.6) years which range from 24-89 years. Two hundred nineteen (56%) of the respondents were females, 215 (55%) were currently married, and two third (66.2%) of the respondents were orthodox Christian followers. One fourth (25.8%) were Colleague/university, and 316(80.8%) of the respondents were urban dwellers (Table 1). About half (47.3%) of the respondents had poor knowledge on hypertension.

**Table 1:** socio-demographic characteristics of uncontrolled hypertension among adult hypertensive patients on follow up at Mekelle city public hospitals, Tigray, Northern Ethiopia, 2019

Variables	Frequency	Percent
**Sex**		
Female	219	56.0
Male	172	44.0
**Age**		
<50 year	325	83.1
>=50 year	66	16.9
**Marital status**		
Single	29	7.4
Married	215	55.0
Divorced	85	21.7
Widowed	62	15.9
**Religion**		
Orthodox	259	66.2
Muslim	109	27.9
Catholic	17	4.3
Protestant	6	1.5
**Educational status**		
No formal education	117	29.9
1-8	74	18.9
9-12	99	25.3
Colleague/university	101	25.8
**Occupation**		
Governmental employee	105	26.9
Private employee	39	10.0
Merchant	111	28.4
Farmer	51	13.0
Others*	85	21.7
**Residency**		
Rural	75	19.2
Urban	316	80.8

Others* in occupation means student, unemployed and pensioner

**Behavioral characteristics of respondents:** of all respondents, one fourth (26.1%), 231(59.1%), 289(73.9%), and 151(38.6%) were non adherent to anti-hypertensive medication, physical exercise, low salt diet, and weight management respectively. About three fourth (76%) of the participants were adherent to alcohol abstinence (Table 2).

**Table 2:** behavioral characteristics of uncontrolled hypertension among adult hypertensive patients on follow up at Mekelle city public hospitals, Tigray, Northern Ethiopia, 2019

Variables	Frequency	Percent
**Anti-hypertensive medication adherence**		
Non-adherent	102	26.1
Adherent	289	73.9
**Adherence to physical exercise**		
Non-adherent	231	59.1
Adherent	160	40.9
**Cigarette smoking status**		
No	385	98.5
Yes	6	1.5
**Are you taking alcohol?**		
No	297	76.0
Yes	94	24.0
**Adherence to low salt diet**		
Non-adherent	289	73.9
Adherent	102	26.1
**Adherence to weight management**		
Non-adherent	151	38.6
Adherent	240	61.4

**Health profile characteristics:** the mean duration of hypertension was 5.39 years (± 3.76) years with a minimum of 1 year and a maximum of 25 years. Of all respondents, 75(19.2%) had comorbid disease. From those who had comorbid disease, 64(85.3%), and 10(13.3%) had DM and CVD respectively. Among the respondents, 52(13.3%) of the participants reported as they had family history of hypertension, and 318(81.3%) of the study subjects had been taking one type of drugs per day (Table 3).

**Table 3:** health profile related characteristics of uncontrolled hypertension among adult hypertensive patients on follow up at Mekelle public hospitals, Tigray, Ethiopia, 2019

Variables	Frequency	Percent
**Duration of hypertension**		
<5 years	197	50.4
5-10 years	170	43.5
>10 years	24	6.1
**Family history of hypertension**		
No	339	86.7
Yes	52	13.3
**Do you miss your follow up**		
No	337	86.2
Yes	54	13.8
**BP cuff at home**		
No	328	83.9
Yes	63	16.1
**BMI status**		
Under weight	15	3.8
normal weight	307	78.5
Over weight	57	14.6
Obese	12	3.1
**Comorbid diseases**		
No	316	80.8
Yes	75	19.2
**Diabetes**		
No	11	14.7
Yes	64	85.3
CVD		
No	65	86.7
Yes	10	13.3
**Number of medications**		
Mono therapy	318	81.3
Bi therapy	52	13.3
≥ 3 therapy	21	5.4

**Uncontrolled hypertension and associated factors:** the magnitude of uncontrolled hypertension was found to 48.6 % (95%CI: 43.5-53.2%). In the bivariate logistic regression analysis, uncontrolled hypertension was significantly associated with nine variables. After adjustment for potential confounders older age, non-adherence to antihypertensive medication, non-adherence to physical exercise, non-adherence to dietary management and non-adherence to weight management were significantly and positively associated with uncontrolled hypertension among hypertensive patients. The odds of having uncontrolled hypertension was 2.3 times higher among the age of ≥ 50 years old compared to the age of < 50 years old (AOR = 2.33, 95%CI: 1.25,4.35). Patients who were non-adherent to their prescribed antihypertensive drugs were two times (AOR = 1.82 95%CI: 1.08, 3.04) more likely to have uncontrolled hypertension as compared to those who were adherent to their antihypertensive drugs. Patients who didn’t adhere to physical exercise were 1.8 times more likely to have uncontrolled hypertension compared to those who adhered to physical exercise (AOR = 1.79 95%CI: 1.13, 2.83). Hypertensive patients who were non-adherent to Low-salt diet were two times more likely to develop uncontrolled hypertension compared to counterparts (AOR = 1.98 95%CI: 1.18, 3.31). Similarly, the odds of having uncontrolled hypertension were two times higher among non-adherent to weight management respondents compared to adherent respondents (AOR = 2.06, 95%CI: 1.31,3.23) (Table 4).

**Table 4:** logistic regression analysis of factors associated with uncontrolled hypertension among adult hypertensive patients on follow up at Mekelle city public hospitals, Tigray, Ethiopia, 2019

Variable	Blood pressure control		COR (95% CI)	AOR (95% CI)	P-value
	Controlled	Uncontrolled			
Sex					
Female	124 (56.6%)	95 (43.4%)	1	1	
Male	77 (44.8%)	95 (55.2%)	1.6 (1.077,2.41)	1.36 (0.88,2.1)	0.17
Age					
< 50 years	182 (56%)	143 (44%)	1	1	
>= 50 years	19 (28.8%)	47 (71.2%)	3.15 (1.77,5.6)	2.33 (1.25,4.35)	0.008
Anti HTN medication Adherence					
Non-adherent	36 (35.3%)	66(64.7%)	2.44 (1.53,3.9)	1.82 (1.08,3.04)	0.024
Adherent	165 (57.1%)	124(42.9%)	1	1	
Adherence to physical exercise					
Non-adherent	98 (42.2%)	133 (57.6%)	2.45 (1.62,3.72)	1.79 (1.13,2.83)	0.013
Adherent	103 (64.4%)	57 (35.6%)	1	1	
Low-salt diet adherence					
Non-adherent	135 (46.7%)	154 (53.3%)	2.09 (1.31,3.34)	1.98(1.18,3.31)	0.01
Adherent	66 (64.7%)	36 (35.3%)	1	1	
Adherence to Weight management					
Non-adherent	61(40.4%)	90(59.6%)	2.07 (1.365,3.13)	2.06 (1.31,3.23)	0.002
Adherent	140(58.3%)	100(41.7%)	1	1	
BP cuff at home					
No	173(52.7%)	155(47.3%)	1.4 (.81,2.4)	1.485 (.82,2.7)	0.195
Yes	28(44.4%)	35(55.6%)	1	1	
Comorbid disease					
No	171(54.1%)	145(45.9%)	1	1	
Yes	30(40%)	45(60%)	1.77 (1.06,2.95)	1.516 (.85,2.704)	0.16
Number of medications					
Mono therapy	165(51.9%)	153(48.1%)	1	1	
Bi therapy	29(55.8%)	23(44.2%)	.86 (.474,1.54)	1.03 (.546,1.93)	0.94
≥ 3 therapy	7(33.3%)	14(66.7%)	2.16 (.85,5.49)	1.27 (.45,3.55)	0.65
Hosmer and Lemeshow Test=0.741					

## Discussion

This study revealed that 48.6% (95% CI: 43.5-53.2%) of hypertensive patients had uncontrolled hypertension. This finding is in line with studies done Lebanon (51.1%) [17], Malaysia (51.7%) [26], in Kwazulu-Natal (51%) [27], Jimma hospital Ethiopia (52.7%) [13], and Ayder comprehensive specialized hospital Ethiopia (51.2%) [20]. It was lower than studies done in Southern China (55.4%), Western India 63.6% [28], Panama (66.7%) [29], South Africa (75.5%) [30], Kinshasa (77.5%) [31], Cameroon (63.2%) [32], Zimbabwe (67.2%) [33], Ethiopia (63%) [12] and Zewditu Ethiopia (69.9%) [11], and Debre Tabor, Northwest Ethiopia (57.1%) [24]. However, the magnitude of uncontrolled hypertension in this study was higher than the finding of previous studies done in Israel (35.9%) [34], Sudan (36%) [35], and university of Gondar hospital Ethiopia (37%) [10]. This discrepancy could be due to the difference in drug adherence level, study population, degree of urbanization, differences in lifestyle behaviors, dietary habits, and environmental factors. In this study, age ≥ 50 years old was significantly and positively associated with uncontrolled hypertension compared to age < 50 years old. This finding also agrees with studies done in India [21], Morocco [36], Uganda [23], Angola [37], and Jimma Ethiopia [13]. This is due to aging causes loss of elasticity of vasculature, arterial stiffening, which in turn leads to peripheral vascular resistance and uncontrolled hypertension [38, 39]. Antihypertensive medication non-adherence was another factor significantly associated with uncontrolled hypertension. Hypertensive patients who were non-adherent to their prescribed antihypertensive drugs were nearly two times more likely to have uncontrolled hypertension as compared to those who were adherent to their prescribed antihypertensive drugs. This finding is supported by studies done in Ghana, University of Gondar hospital Ethiopia, and Ayder comprehensive specialized hospital Ethiopia [10, 20, 40]. Other studies done in Malaysia [41], Southern California [42], South Asia [43], Cameron [32], and South Africa [44] revealed that good adherence to antihypertensive medication was found a preventive factor to uncontrolled hypertension. This might be due to good adherence to antihypertensive medications is crucial to lower high blood pressure through vasodilatation, increase urination which reduces sodium and fluid in the body and blocking of the sympathetic activation of the heart [45]. Non-adherence to physical activity and non-adherent to weight management were statistically and positively associated with uncontrolled hypertension. Hypertensive patients who didn’t adhere to physical exercise were 1.8 times more likely to have uncontrolled hypertension compared to those who adhered to physical exercise. This finding is supported by the previous studies done in China, sub-Saharan countries, Debre Tabor Ethiopia and Ayder Ethiopia [1, 20, 22, 24]. This may be due to adherence to physical activity controls high blood pressure through enhancement of renal function (decreasing of cardiometabolic risk factors), and preventing weight gain [46, 47]. Similarly, Non-adherence to a low-salt diet was found significantly associated with uncontrolled hypertension. This is consistent with studies done in Macau China, Southern China, and Ethiopia [22, 24, 48]. This may be due to the effect of high-salt diets on the function of the renin-angiotensin system that causes fluid retention which increases the cardiac burden and uncontrolled hypertension [49].

## Conclusion

The prevalence of uncontrolled hypertension among adult hypertensive patients was high. This study revealed that nearly one out of two hypertensive patients had uncontrolled hypertension. Older age, non-adherence to antihypertensive medication, non-adherence to physical exercise, non-adherence to low salt diet and non-adherence to weight management were significantly associated with uncontrolled hypertension. Therefore, more effort should be dedicated to those identified modifiable risk factors to maximize blood pressure control.

### What is known about this topic

- Uncontrolled hypertension has become the commonest cause of cardiovascular, renal, and cerebrovascular morbidities and mortalities on the continent;
- The prevalence of uncontrolled hypertension among adult hypertensive patients in Africa is a major public health problem;
- The annual death of Ethiopia population due to non-communicable disease such as uncontrolled hypertension is still high (39%).

### What this study adds

- The prevalence of uncontrolled hypertension among adult hypertensive patients was considerably high;
- Older age, non-adherence to antihypertensive medication, physical inactivity, non-adherence to low salt diet and non-adherence to weight management were significantly associated with uncontrolled hypertension;
- Health care personnel should be concerned to those identified modifiable risk factors to maximize blood pressure control.
